# Two Pathogenic Gene Mutations Identified Associating with Congenital Cataract and Iris Coloboma Respectively in a Chinese Family

**DOI:** 10.1155/2020/7054315

**Published:** 2020-02-19

**Authors:** Bin Li, Bin Lu, Xuewen Guo, Shenghui Hu, Guihu Zhao, Weihong Huang, Jianzhong Hu, Kun Song

**Affiliations:** ^1^National Clinical Research Center for Geriatric Disorders, Xiangya Hospital, Central South University, Changsha, China; ^2^Mobile Health Ministry of Education—China Mobile Joint Laboratory, Xiangya Hospital, Central South University, Changsha, China; ^3^Department of Pathogen Biology, School of Basic Medical Sciences, Central South University, Changsha, China; ^4^Department of Neurology, Liaocheng Dongchangfu People's Hospital, Liaocheng, China; ^5^Department of Orthopaedics, Xiangya Second Hospital, Central South University, Changsha, China; ^6^Department of Spine Surgery, Xiangya Hospital, Central South University, Changsha, China; ^7^Department of Gastrointestinal Surgery, Xiangya Hospital, Central South University, Changsha, China

## Abstract

**Purpose:**

To screen out pathogenic genes in a Chinese family with congenital cataract and iris coloboma. *Material and Methods*. A three-generation family with congenital cataract and iris coloboma from a Han ethnicity was recruited. DNA was extracted from peripheral blood samples collected from all individuals in the family. Whole exon sequencing was employed for screening the disease-causing gene mutations in the proband, and Sanger sequencing was used for other members of the family and a control group of 500 healthy individuals. Bioinformatics analysis and three-dimensional structure predictions were used to predict the impact of amino acid changes on protein structure and function.

**Results:**

The candidate genes of cataract and iris coloboma were successfully screened out. A heterozygote mutation, *CRYGD* c.70C>A (p.P24T), was identified as cosegregating with congenital cataracts, while another heterozygous mutation, *WFS1* c.1514G>C (p.C505S), which had not been reported previously, cosegregated with congenital iris coloboma. Bioinformatic analyses and three-dimensional structure prediction proved that the three-dimensional structures of *WFS1* c.1514G>C (p.C505S), which had not been reported previously, cosegregated with congenital iris coloboma. Bioinformatic analyses and three-dimensional structure prediction proved that the three-dimensional structures of *CRYGD* c.70C>A (p.P24T), was identified as cosegregating with congenital cataracts, while another heterozygous mutation,

**Conclusions:**

We report a novel mutation, *WFS1* p.C505S, and a known mutation, *CRYGD* p.P24T, that cosegregate with iris coloboma and congenital cataract, respectively, in a Chinese family. This is the first time the association of *WFS1* p.C505S with iris coloboma has been demonstrated, although *CRYGD* p.P24T has been widely reported as being associated with congenital cataract, especially in the Eastern Asian population. These findings may have future therapeutic benefit for the diagnosis of iris coloboma and congenital cataract. The results may also be relevant in further studies aiming to investigate the molecular pathogenesis of iris coloboma and congenital cataract.*WFS1* c.1514G>C (p.C505S), which had not been reported previously, cosegregated with congenital iris coloboma. Bioinformatic analyses and three-dimensional structure prediction proved that the three-dimensional structures of *CRYGD* c.70C>A (p.P24T), was identified as cosegregating with congenital cataracts, while another heterozygous mutation, *WFS1* c.1514G>C (p.C505S), which had not been reported previously, cosegregated with congenital iris coloboma. Bioinformatic analyses and three-dimensional structure prediction proved that the three-dimensional structures of *CRYGD* c.70C>A (p.P24T), was identified as cosegregating with congenital cataracts, while another heterozygous mutation,

## 1. Introduction

Congenital cataract complicated by iris coloboma is not clinically common. Congenital cataract (OMIM 604307), which is an opacification of the ocular lens present at birth [1], is usually hereditary and can be transmitted as a dominant or a recessive trait. Congenital cataract is a major cause of visual impairment and blindness worldwide, with at least a third of which being familial, and accounts for 10% of all childhood blindness [2–4]. The primary consequences of lens cloudiness include glare, contrast loss, and decrease in vision that eventually lead to blindness. Various etiological factors have been identified, including infection, metabolic disorders, and genetic defects [5]. Iris coloboma is a special phenotype of congenital aniridia, defined as a rare bilateral preocular disorder characterized by the complete or partial absence of the iris [6]. Iris coloboma is most commonly inherited as an autosomal-dominant disorder [6]. It is an anatomical defect generated as a result of defective embryogenesis in ocular structures [7]. If the choroidal fissure is not completely closed at the iris, the defect will exist under the iris, and the round pupil will appear as a key hole shape known as iris coloboma. The prevalence range is approximately 1 : 64,000 to 1 : 96,000 worldwide, and approximately 1 : 100,000 in China [8].

Congenital cataracts are highly heterogeneous clinically and show interfamilial and intrafamilial variability [9]. At present, at least 45 genes of nonsyndromic congenital cataract with Mendelian inheritance have been reported [10–12]. ([Supplementary-material supplementary-material-1]). The inheritance modes of these genes include autosomal dominant (AD), autosomal recessive (AR), and X-linked recessive (XR), but autosomal dominant is the main inheritance mode [13]. These pathogenic genes can be divided into six categories: lens protein gene, membrane protein gene, cytoskeleton protein gene, developmental regulatory protein gene, tyrosine kinase receptor gene, and chromatin-modified protein gene, in addition to some syndrome-related genes related to cataract [14]. Among them, lens protein genes, such as *CRYAA*, *CRYAB*, and *CRYGD*, encode nearly 50% of the protein in the lens [14]. About two-thirds of the cases of congenital iris coloboma are inherited as an autosomal-dominant trait with genetic heterogeneity [15–17]. These genes have been mapped on chromosome 11p13 by linkage analysis and positional cloning. Approximately 80% of congenital iris coloboma cases are caused by the gene mutation of human paired box-6 (*PAX6*) [8].

This study aims to identify the pathogenic genes of cataract and iris coloboma in a family by whole exon sequencing combined with cosegregation analysis, identifying the mutation sites and performing informatic analysis to preliminarily detect the possible genetic factors of the disease, and to find strategies and methods for the treatment of cataract and iris coloboma.

## 2. Materials and Methods

### 2.1. Subject Recruitment and Clinical Examination

A family with congenital cataracts from a Han ethnicity was recruited for the present study. In total, 14 subjects were enrolled and underwent detailed clinical examinations. All members of the family were diagnosed by the Aier Eye Hospital in Hunan province. Four individuals were affected by cataracts, and three were diagnosed with iris coloboma with or without cataract. Peripheral blood samples were collected from all the survivors of the family after informed consent was obtained from all members. This study was approved by the Institutional Review Board of the Xiangya Hospital of Central South University (Changsha, China) and performed in accordance with the Declaration of Helsinki.

### 2.2. Genomic DNA Preparation and High Throughput Sequencing

The genomic DNA of peripheral blood lymphocytes of the probands was extracted by the phenol-chloroform method. DNA concentration and purity were detected by NanoDrop 1000 ultramicro spectrophotometer. Ultrasound degradation was used to break 5µg genomic DNA into 150–200 bp fragments, and junctions were added at both ends of the fragments to construct the library. The constructed library was amplified via polymerase chain reaction (PCR). The reaction system of library amplification was prepared on ice, and the reaction program was set up to carry out PCR on the PE-2720 PCR instrument. The thermal cycle conditions for PCR are as follows: initial denaturation at 95°C for 2 min, followed by 30 cycles of denaturation at 95°C for 30 sec, annealing at 65°C for 30 sec and elongation at 72°C for 1 min, cycling 4–6 times, and a final extension at 75°C for 10 min. The library capture mixture was prepared, the hybrids were captured, and the obtained hybrid library was further amplified by PCR. Finally, the hybrid library was sequenced by Illumina HiSeq 2000 system.

### 2.3. Bioinformatics Analysis

We evaluated the sequence quality and aligned the reads to the human reference genome (GRCh37/hg19) using a Burrows-Wheeler Aligner. Single nucleotide variations (SNVs) and insertions and deletions (Indels) were called by the genome analysis toolkit. The candidate variants were filtered according to the following rules: (1) eliminate variation in noncoding regions and synonymous variation in coding regions; (2) exclude nonrare (MAF ≥0.01) variants in the 1000 Genomes Project and ESP Exon Project; (3) exclude the mutation in the sequencing data of the normal control exomes already in the laboratory.

### 2.4. Sanger Sequencing Analysis

In order to confirm whether the disease phenotype is cosegregated with candidate genes, Sanger sequencing was used to confirm that the two mutations were not detected in the unaffected family members and in another 500 unrelated controls. Peripheral blood was collected, and genomic DNA was extracted from blood lymphocytes and leukocytes by the standard phenol-chloroform method. Sanger sequencing of all the individuals was performed following the standard procedures.

### 2.5. Functional Prediction of Proteins

Twenty-four software programs and arithmetics, including SIFT, LRT, MutationTaster, FATHMM, PROVEAN, VEST3, MetaSVM, MetaLR, M-CAP, FATHMM-MKL, Eigen, GenoCanyon, fitCons, REVEL, ReVe, GERP++, phyloP, phastCons, SiPhy, CADD, DANN, MutationAssessor, PolyPhen-2 HVAR, and PolyPhen-2 HDIV in VarCards (http://159.226.67.237/sun/varcards/welcome/index) [18], were employed to predict the harmfulness of mutated protein. I-TASSER (http://zhanglab.ccmb.med.umich.edu/I-TASSER/) and PyMol-1.5.x software were used to predict and visualize the three-dimensional (3D) model of the proteins. Additionally, the 3D model of reported mutants, which were in the same domain or in the same functional structures in the gene, was also predicted for contrastive analysis.

## 3. Results

### 3.1. Clinical Evaluation

**Figure 1:**
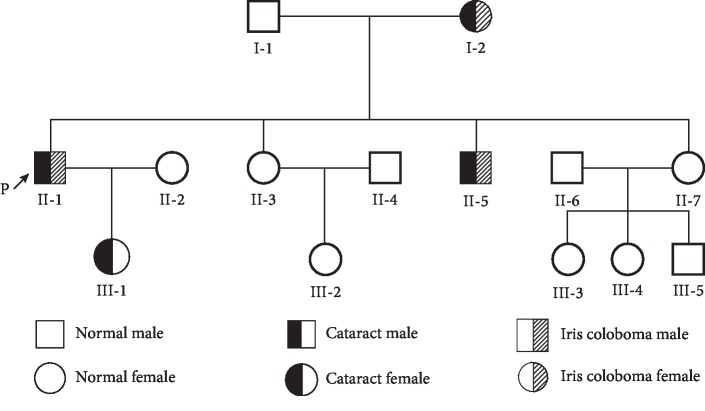
Family diagram. The pedigree of the congenital cataract with iris coloboma: circles represent women; squares represent men. Black indicates cataract patients, gray shadows indicate iris coloboma patients, and the arrow indicates the proband (II:1).

**Figure 2:**
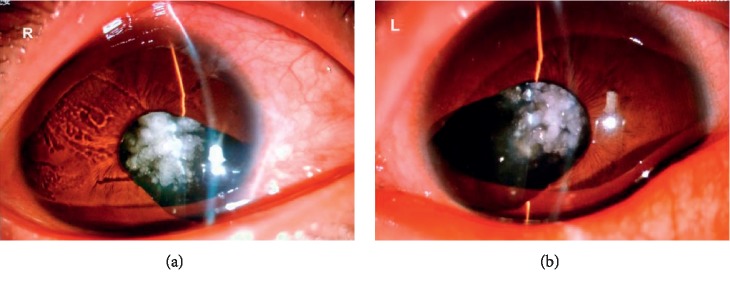
Proband (II:1). Photograph of the anterior segment of the slit lamp. It can be seen that the bilateral cornea of the patient is clear, the pupil is not round, the iris is missing, and the central cauliflower pattern is cloudy.

A three-generation family with diagnosed cataract with iris coloboma was recruited for this study. The family consists of 14 individuals, including 6 males and 8 females (Figure 1). The slit lamp clinical examination of anterior segment revealed the proband (II:1) with congenital cataract accompanying iris coloboma (Figure 2). Patients I:2, II:1, II:5, and III:1 were diagnosed as having congenital cataracts, and patients I:2, II:1, and II:5 had accompanying iris coloboma simultaneously.

### 3.2. Mutation and Cosegregation Analyses

Sequence assembly with BWA and SAMtools was performed to obtain the genotype of each gene locus, and then detection and annotation classification of SNP and indels were performed by SAMtools and a genome analysis toolkit (GATK). Samples from the family were found to have 21,595 mutation sites containing 11,299 nonsynonymous mutations, splice junction mutations, and insertional deletion mutations. A total of 216 mutations with a frequency of less than 0.01 in the population were screened out from the 1000 Genomes database. Six mutation sites of five related genes (BCOR, FYCO1, SIX6, *WFS1*, and *CRYGD*) were screened by comparison with data provided by ClinVar.

**Figure 3:**
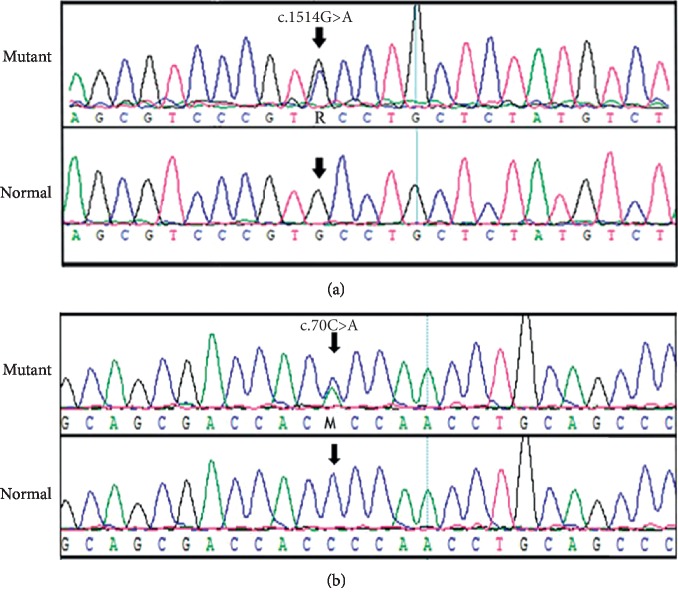
Mutant screening sequencing alignment diagram for family. (a) The sequence chromatogram (forward strand) shows a heterozygous c.1514G>A transition that causes a substitution of serine (S) for cysteine (C) at codon 505. The arrow points to the position of the mutant nucleotide. (b) The sequence chromatogram (forward strand) shows a heterozygous c.70C>T transition that causes a substitution of threonine (T) for proline (P) at codon 24. The arrow points to the position of the mutant nucleotide.

A heterozygous mutation c.70C>A in the *CRYGD* gene and a heterozygous mutation c.1514G>C in the *WFS1* gene were detected in the proband II:1 (Figure 3). In addition, the *CRYGD* c.70C>A mutation was screened out in another three cataract patients (I:2, II:5 and III:1), and *WFS1* c.1514G>C was screened out in another two iris coloboma patients (I:2, and II:5) (Figure 1). Neither *CRYGD* c.70C>A nor *WFS1* c.1514G>C was detected in the healthy individuals in this lineage or in the other 500 healthy patients. It was thus confirmed that *CRYGD* c.70C>A and *WFS1* c.1514G>C cosegregate with congenital cataract and iris coloboma, respectively.

### 3.3. Bioinformatics Analysis

Because the heterozygous c.70C>T (p.P24T) in *CRYGD* has been recognized as the cataract-causing gene mutation, [2, 19–24] we performed functional prediction only for *WFS1* c.1514G>C. In our results, 19 of the 24 functional prediction programs in VarCards showed that c.1514G>C in *WFS1* was possibly harmful (Table 1).

**Figure 4:**
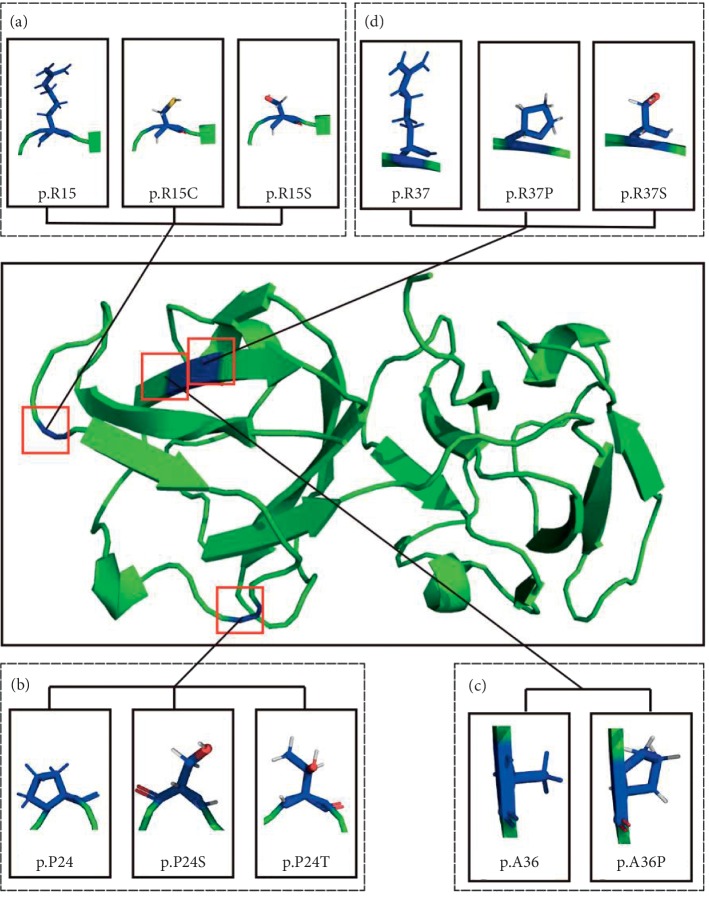
The 3D conformation of seven reported pathogenic mutation sites located in the first “Greek Key” module domain of *CRYGD*, as predicted by modeling software. (a) The 3D conformation of the wild type *CRYGD* 15th amino acid arginine and mutant cysteine and serine. (b) The 3D conformation of the wild type *CRYGD* 24th amino acid proline and the mutant threonine and serine. (c) The 3D conformation of the wild type *CRYGD* 36th amino acid alanine and the mutant Proline. (d) The 3D conformation of the wild type *CRYGD* 37th amino acid arginine and mutant serine and proline. 3D, three-dimensional.

3D structure prediction software was used to reveal the difference between the wild type and the mutant type in *CRYGD* and *WFS1*. The wild type of *CRYGD* p.P24 exhibited a pyrrole ring structure, while the p.P24T mutant changed into a hydrophilic structure (Figure 4). We studied another six reported mutation sites located in the first “Greek Key” module domain of *CRYGD* protein as having the p.P24T mutant, such as p.R15C, p.R15S, p.P24S, p.A36P, p.R37P, and p.R37S, which have been reported to be associated with cataracts. According to the results of 3D structure prediction, compared with the wild type *CRYGD*, all the three mutants, p.R15C, p.R15S, and p.R37S, changed structure noticeably from the long side to the short side chains, while p.A36P and p.R37P exhibited proline with pyrrole rings in comparison to the wild type. However, in mutants p.P24S and p.P24T the original pyrrole rings did not appear in the wild type protein (Figure 4).

**Figure 5:**
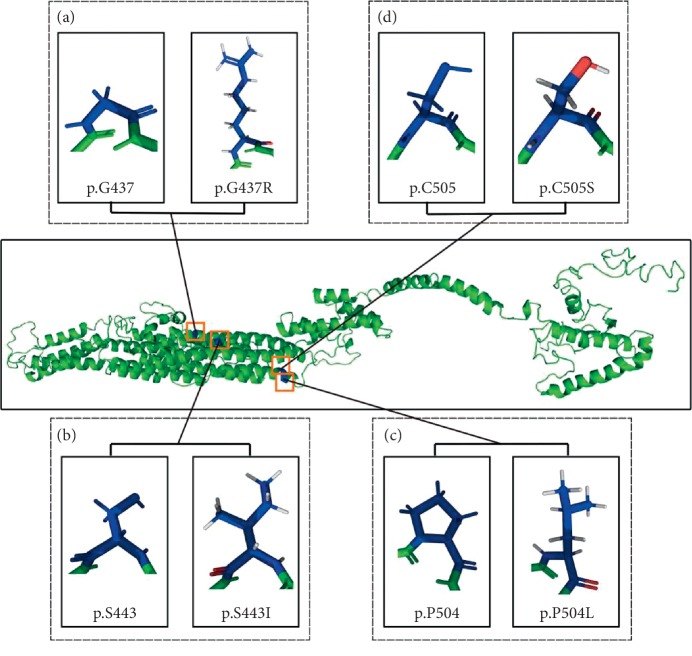
The 3D conformation of four mutation sites located in the transmembrane domain of *WFS1*, as predicted by modeling software. (a) The 3D conformation of the wild type *WFS1* 437th amino acid glycine and mutant arginine. (b) The 3D conformation of the wild type *WFS1* 443rd amino acid serine and the mutant isoleucine. (c) The 3D conformation of the wild type *WFS1* 504th amino acid proline and the mutant leucine. (d) The 3D conformation of the wild type *WFS1* 505th amino acid cysteine and mutant serine. 3D, three-dimensional.

Moreover, three reported disease-causing mutations in *WFS1*, including p.G437R, p.S443I, and p.P504L, located in the transmembrane domain as with p.C505S, were 3D structures predicted as requiring further analysis. In that analysis, p.P504L did not exhibit original pyrrole rings compared with p.P504, while p.G437R, p.S443I, and p.P504L underwent much greater changes than the wild type (Figure 5).

## 4. Discussion

With the development of exon sequencing technology and its decreasing cost, it has been widely used in screening for disease-causing genes. In this study, the candidate genes for cataract and iris coloboma were successfully screened by exon sequencing in the probands with cataract and iris coloboma. By Sanger sequencing, a heterozygote mutation, c.70C>A (p.P24T) in *CRYGD*, was screened out in all cataract patients and was not detected in noncataract patients. A heterozygous mutation, c.1514G>C (p.C505S) in *WFS1*, was screened in all iris coloboma patients and was absent in subjects without iris coloboma. In addition, these mutations were absent in the other 500 random healthy individuals. It was thus confirmed that *CRYGD* c.70C>A and *WFS1* c.1514G>C cosegregate with the disease phenotypes examined. It is also preliminarily confirmed that these two gene mutations are responsible for congenital cataract and iris coloboma, respectively.

Literature review shows that cataracts caused by the mutation in *CRYGD* had been reported in many published articles since the 1990s and that the biological function of the mutant protein was deeply and meticulously researched in many papers [2, 21, 22, 25–27]. The p.P24T mutation in *CRYGD* has been widely reported as the cause of congenital cataract, especially in the East Asian population [2, 19–24]. The *CRYGD* gene encodes *γ*D-crystallin, the lens protein, with 174 amino acids [28, 29]. *γ*D-crystallins are monomer proteins whose polypeptide chains fold into four similar “Greek key” motifs. The last two of the “Greek key” modules form a domain joined by connecting fragments [30, 31]. At least eight mutations related to congenital cataract have been reported in the lens protein gene *CRYGD* [2, 21, 22, 26, 27, 32]. These mutations can change the structure or three-dimensional conformation of lens protein, affecting its solubility, stability, or orderliness, leading to cataracts [2, 21, 22, 26, 27]. In this study, we analyzed the structure of p.P24T and another six mutation sites in the first “Greek Key” module domain of the *CRYGD* protein, that is, p.R15C, p.R15S, p.P24S, p.A36P, p.R37S, and p.R37P, which have been reported as being associated with cataract.

I-TASSER and PyMol-1.5.x software were used to investigate the 3D structure of the protein and to compare wild type *CRYGD* with the p.P24T mutant. It was revealed that the substitution of proline with threonine changes the structure of the amino acid at position 24 from a pyrrole ring to a nonpyrrole ring (Figure 4). After proline was changed to threonine, there was a branched beta carbon atom in the side chain of the threonine, making the beta-chain tend to extend rather than fold back, which may affect the correct folding of the protein material [2, 33]. Previous studies have shown that the solubility of the p.P24T mutant protein is significantly lower than that of the wild type *CRYGD* protein, which may lead to decreased solubility and the aggregation of polymer material [2, 33].

The p.R15C mutation is located in exon 2 of the *CRYGD* gene and changes a hydrophilic charged amino acid (arginine) to a neutral one (cysteine) (Figure 4). Previous studies showed that the presence of newly exposed cysteine residues in p.R15C mutants would allow the formation of intermolecular disulfide bonds under oxidative conditions, leading to protein aggregation, which is the main feature of many cataracts [34]. In addition, increased hydrophobicity at the p.R15S mutation site may affect protein-protein interactions, since p.R15 is located on a solvent-accessible protein surface [35]. Moreover, the change of p.P24S from hydrophobic proline with pyrrole ring to serine with hydrophilic structure may affect the solubility of *γ*D-crystallin [19, 20, 36]. This effect may be caused by a change in the hydrogen binding characteristics of the protein-water interface [19, 20, 36]. Regarding p.A36P, it was reported that the mutant structure may affect the normal folding in the tertiary structure of amino acids, leading to decreased solubility, and the accumulation of macromolecule substances may lead to decreased lens transparency, eventually leading to the occurrence of cataract [37]. Moreover, previous studies showed that p.P37S may lead to decreased solubility, and serine residues will destroy the normal beta lamellar structure of proteins, allowing the formation of “beta-bulge” [38], while p.R37P reduced protein solubility by increasing the local hydrophobicity in affected individuals [39].

Congenital iris coloboma is caused by incomplete closure of the fetal cleft at organogenesis [7]. Colobomas of the iris are closure defects of embryonic fissures that can occur in the fifth week of gestation. They often appear in the lower nasal quadrant and can be either isolated or associated with colobomas of the lens, choroid, and optic nerve [40, 41]. Prior to this study, *WFS1* p.C505S had not been reported in association with the iris coloboma phenotype. The *WFS1* gene encodes the transmembrane protein (wolframin) with 890 amino acids containing nine transmembrane domains [42]. *WFS1* was known as the main pathogenic gene of Wolfram syndrome [43], which is an autosomal recessive neurodegenerative disorder characterized by visual symptoms, diabetes mellitus, central diabetes insipidus, and deafness [44]. *WFS1* is also a key element in the endoplasmic reticulum stress activation signaling cascade known as unfolded protein response [45]. Therefore, the mutation of *WFS1* may affect wolframin in regulating the homeostasis of endoplasmic reticulum, thereby causing incomplete closure of the fetal fissure during organogenesis, resulting in iris coloboma. Studies show that changes in the transmembrane domain have a serious impact on the function of wolframin proteins [46]. However, the mutation p.C505S was located in the fifth domain. For further comparison and analysis, p.C505S and three other missense mutations reported to cause disease were predicted by 3D structures in I-TASSER.

The predicted results from 15 functional prediction software programs showed c.1514G>C in *WFS1* was possibly harmful. The novel p.C505S mutation is likely pathogenic for the following reasons: (1) it has never been reported, and it achieved cosegregation with disease phenotypes; (2) it occurs within the highly preserved fifth transmembrane segment, and it is close to the p.P504L mutation, which was previously reported as a disease-causing gene [42]. 3D structure prediction exhibited that p.G437R, p.S443I, and p.P504L underwent greater change than the wild type. The p.G437R mutation, which changed from a neutral hydrophobic glycine to an alkaline hydrophilic arginine, was reported to destroy the normal function of wolframin and lead to Wolfram syndrome in Great Britain [46]. By contrast, p.S443I changed a hydrophilic amino acid into a hydrophobic one. Studies showed that the polarity change caused by p.S443I mutation is likely to interrupt the transmembrane domains of wolframin [47]. Compared to p.P504, the 3D structure of p.P504L exhibited proline with a pyrrole ring to a leucine with nonpyrrole ring (Figure 5). Studies showed that the p.P504L mutation is associated with the onset of Wolfram syndrome [48]. In addition, the 648^th^ amino acid, a reported disease-causing gene located on the transmembrane, was deemed to be an easily truncated variant [46, 49].

Above all, our research provided a reference for the diagnosis of congenital iris coloboma. Meanwhile, we firstly described *WFS1* gene mutation associated with congenital iris coloboma and enriched its gene mutation spectrum, which may also provide a new sight for gene therapy for this disease.

However, our study does have several limitations. First, the sample size was relatively small, only the Chinese population was screened, and, compared with other small size group research, our description of family history and clinical symptoms is not comprehensive enough [50–52]. In addition, some research indicated that the mutation of *WFS1* may also cause cataract, so we cannot exclude the possibility that the joint action of the *CRYGD* and *WFS1* cause cataract and iris coloboma [37, 53, 54]. Second, although some software has been used to predict mutations, it has not been validated by molecular, cellular, and animal experiments. Finally, although we have done a lot of research on the *WFS1* protein, the exact molecular function of the *WFS1* protein in the development of the iris remains unknown.

## 5. Conclusion

In conclusion, a novel *WFS1* p.C505S mutation and a *CRYGD* p.P24T mutation were found in a family with cataract and iris coloboma, and they were cosegregated from iris coloboma and cataract, respectively. Structural analysis in this study also highlighted the unique role of a single amino acid in the change of protein properties. It was proved that the C505S mutation of *WFS1* contributes greatly to the occurrence of iris coloboma and corroborated that the *CRYGD* p.P24T possibly leads to cataract, although *CRYGD* p.P24T has been detected in East Asia widely and identified as having a biological function in cataracts. Further exploration of the pathogenesis of the disease is needed, as it also plays a major role in prevention and treatment of the disease. We expect that more researchers will take up exon sequencing combined with bioinformatics analysis as a major scientific method and diagnostic tool.

## Figures and Tables

**Table 1:** Functional prediction results by twenty-four software programs and arithmetics.

Gene symbol	*WFS1*
Nucleotide change	c. 1514G>C
Exon	8
Amino acid change	p.C505S
Domain	Fifth transmembrane domain
SIFT	Damaging
LRT	Deleterious
Mutation taster	Disease-causing
FATHMM	Damaging
PROVEAN	Damaging
VEST3	Damaging
MetaSVM	Damaging
MetaLR	Damaging
M-CAP	Damaging
FATHMM-MKL	Damaging
Eigen	Damaging
GenoCanyon	Damaging
fitCons	Damaging
REVEL	Damaging
ReVe	Damaging
GERP++	Conserved
phyloP	Conserved
phastCons	Conserved
SiPhy	Conserved
CADD	Tolerable
DANN	Tolerable
MutationAssessor	Medium
PolyPhen-2 HDIV	Benign
PolyPhen-2 HVAR	Benign

## Data Availability

The data used to support the findings of this study are included within the article.

## Supplementary Materials

Supplementary Materials Table S1 reports 45 genes of nonsyndromic congenital cataract with Mendelian inheritance. Genes with widely reported mutations that are associated congenital cataract were listed with gene symbol, nucleotide change, amino acid change, clinical phenotype, and reference.
