# Supplementary material for: Two Pathogenic Gene Mutations Identified Associating with Congenital Cataract and Iris Coloboma Respectively in a Chinese Family
Source: J Ophthalmol. 2020 Feb 19;2020:7054315. doi: 10.1155/2020/7054315 (PMC7049832; doi:10.1155/2020/7054315)
Supplement: Supplementary Materials — Table S1 reports 45 genes of nonsyndromic congenital cataract with Mendelian inheritance. Genes with widely reported mutations that are associated congenital cataract were listed with gene symbol, nucleotide change, amino acid change, clinical phenotype, and reference. [file 7054315.f1.pdf]

Supplementary materials

Gene symbol	Nucleotide change	Amino acid change	Clinical phenotype	Ref
CRYAA	c.35G>T	p. R12L	lens protein gene	[1]
CRYAB	c.32G>A	p. R11H	lens protein gene	[2]
CRYBA1	c.279-281delGAG	p.ΔG91	lens protein gene	[3]
CRYBA4	c.206T>C	p. L69P	lens protein gene	[4]
CRYBB1	c.658G>T	p. G220X	lens protein gene	[5]
CRYBB2	c.563G>A	p. R188H	lens protein gene	[6]
CRYBB3	c.314G>A	p. R105Q	lens protein gene	[7]
CRYGA	c.196T>C	p. Y66H	lens protein gene	[8]
CRYGB	c.449G>T	p. G150V	lens protein gene	[8]
CRYGC	c.385G>T	p. G129C	lens protein gene	[9]
CRYGD	c.70C>A	p. P24T	lens protein gene	[10]
CRYGS	c.53G>T	:p.G18V	lens protein gene	[11]
GJA3	c.188A>G	p.N63S	membrane protein gene	[12]
GJA8	c.262C>T	p.P88S	membrane protein gene	[13]
BFSP1	c736-1384_c.957-66del	T246fsX7	cytoskeleton protein gene	[14]
BFSP2	c.1091G>A	p.G364D	cytoskeleton protein gene	[15]
PAX6	c.307C>T	p.R103X	developmental regulatory protein gen	[16]
PITX3	c.38G>A	p.S13N	developmental regulatory protein gen	[17]
HSF4	c.524G>C	p.R175P	developmental regulatory protein gen	[18]
MAF	c.863G>C	p.R288P	developmental regulatory protein gen	[19]
CHMP4B	c.481G>A	p. E161K	chromatin modified protein gene	[20]
EPHA2	c.2842G>T	p. G948W	tyrosine kinase receptor gene	[21]
COL4A1	c.2345G>C	p. G782A	syndrome-related genes	[22]
FTL	c.160G>A	p.E54K	developmental regulatory protein gen	[23]

GALK1	c.416T>C	p. L139P	syndrome-related genes	[24]
FYCO1	c.808C>T	p. Q270X	chromatin modified protein gene	[25]
NHS	c.2739del	p(F913Lfs*9)	syndrome-related genes	[25]
PEX11B	c.235C>T	p. R79T	syndrome-related genes	[26]
BCOR	c.4706dup	p. (G1570Rfs*7)	syndrome-related genes	[25]
TDRD7	c.688_689insA	P. Y230X	developmental regulatory protein gen	[27]
LSS	c.1741T>C	W581R	syndrome-related genes	[28]
IARS2	c.607G>C	p. G203R	syndrome-related genes	[25]
PXDN	c.2638C>T	p. R880C	syndrome-related genes	[29]
P3H2	c.13C>T	p.Q5X	developmental regulatory protein gen	[30]
FOXE3	c.959G>C	p.X320S	developmental regulatory protein gen	[31]
MYH9	c.3493C>T	p. R1165C	syndrome-related genes	[32]
OCRL	c.1964A>T	p. D655V	syndrome-related genes	[33]
GCNT2	c.1091T>C	p.F364S	syndrome-related genes	[34]
LEMD2	c.38T>G	p.L13R	syndrome-related genes	[35]
FAM126A	c.169T>C	p.C57R	syndrome-related genes	[36]
AGK	c.421G>T	p.E141X	syndrome-related genes	[37]
VIM	c.596G>A	p.E151K	syndrome-related genes	[38]
MIP	c.572C>G	p. P191R	membrane protein gene	[39]
CHMP4B	c.170A>G	p.H57R	syndrome-related genes	[40]
LIM2	c.461G>A	p. G154E	membrane protein gene	[41]

Table S1. Reported 45 genes of non-syndromic congenital cataract with Mendelian inheritance. Genes with widely reported mutations that are associated congenital cataract, were listed with *Gene symbol*,*Nucleotide change*, *Amino acid change*, *Clinical phenotype*, *reference*.

Reference

1. Litt M, Kramer P, LaMorticella DM, Murphey W, Lovrien EW, Weleber RG. Autosomal dominant congenital cataract associated with a missense mutation in the human alpha crystallin gene CRYAA. Hum Mol Genet 1998;**7**(3):471-4
2. Berry V, Francis P, Reddy MA, et al. Alpha-B crystallin gene (CRYAB) mutation causes dominant congenital posterior polar cataract in humans. Am J Hum Genet 2001;**69**(5):1141-5 doi: 10.1086/324158[published Online First: Epub Date]|.
3. Yang G, Zhai X, Zhao J. A recurrent mutation in CRYBA1 is associated with an autosomal dominant congenital nuclear cataract disease in a Chinese family. Mol Vis 2011;**17**:1559-63
4. Billingsley G, Santhiya ST, Paterson AD, et al. CRYBA4, a novel human cataract gene, is also involved in microphthalmia. Am J Hum Genet 2006;**79**(4):702-9 doi: 10.1086/507712[published Online First: Epub Date]|.
5. Mackay DS, Boskovska OB, Knopf HL, Lampi KJ, Shiels A. A nonsense mutation in CRYBB1 associated with autosomal dominant cataract linked to human chromosome 22q. Am J Hum Genet 2002;**71**(5):1216-21 doi: 10.1086/344212[published Online First: Epub Date]|.
6. Gill D, Klose R, Munier FL, et al. Genetic heterogeneity of the Coppock-like cataract: a mutation in CRYBB2 on chromosome 22q11.2. Invest Ophthalmol Vis Sci 2000;**41**(1):159-65
7. Li D, Wang S, Ye H, et al. Distribution of gene mutations in sporadic congenital cataract in a Han Chinese population. Mol Vis 2016;**22**:589-98
8. Mehra S, Kapur S, Vasavada AR. Polymorphisms of the gamma crystallin A and B genes among Indian patients with pediatric cataract. J Postgrad Med 2011;**57**(3):201-5 doi: 10.4103/0022-3859.85205[published Online First: Epub Date]|.
9. Li XQ, Cai HC, Zhou SY, et al. A novel mutation impairing the tertiary structure and stability of gammaC-crystallin (CRYGC) leads to cataract formation in humans and zebrafish lens. Hum Mutat 2012;**33**(2):391-401 doi: 10.1002/humu.21648[published Online First: Epub Date]|.
10. Gu F, Li R, Ma XX, Shi LS, Huang SZ, Ma X. A missense mutation in the gammaD-crystallin gene CRYGD associated with autosomal dominant congenital cataract in a Chinese family. Mol Vis 2006;**12**:26-31
11. Sun H, Ma Z, Li Y, et al. Gamma-S crystallin gene (CRYGS) mutation causes dominant progressive cortical cataract in humans. J Med Genet 2005;**42**(9):706-10 doi: 10.1136/jmg.2004.028274[published Online First: Epub Date]|.
12. Mackay D, Ionides A, Kibar Z, et al. Connexin46 mutations in autosomal dominant congenital cataract. Am J Hum Genet 1999;**64**(5):1357-64 doi: 10.1086/302383[published Online First: Epub Date]|.
13. Shiels A, Mackay D, Ionides A, Berry V, Moore A, Bhattacharya S. A missense mutation in the

human connexin50 gene (GJA8) underlies autosomal dominant "zonular pulverulent" cataract, on chromosome 1q. Am J Hum Genet 1998;**62**(3):526-32 doi: 10.1086/301762[published Online First: Epub Date]|.
14. Ramachandran RD, Perumalsamy V, Hejtmancik JF. Autosomal recessive juvenile onset cataract associated with mutation in BFSP1. Hum Genet 2007;**121**(3-4):475-82 doi: 10.1007/s00439-006-0319-6[published Online First: Epub Date]|.
15. Ma X, Li FF, Wang SZ, Gao C, Zhang M, Zhu SQ. A new mutation in BFSP2 (G1091A) causes autosomal dominant congenital lamellar cataracts. Mol Vis 2008;**14**:1906-11
16. Dansault A, David G, Schwartz C, et al. Three new PAX6 mutations including one causing an unusual ophthalmic phenotype associated with neurodevelopmental abnormalities. Mol Vis 2007;**13**:511-23
17. Semina EV, Ferrell RE, Mintz-Hittner HA, et al. A novel homeobox gene PITX3 is mutated in families with autosomal-dominant cataracts and ASMD. Nat Genet 1998;**19**(2):167-70 doi: 10.1038/527[published Online First: Epub Date]|.
18. Forshew T, Johnson CA, Khaliq S, et al. Locus heterogeneity in autosomal recessive congenital cataracts: linkage to 9q and germline HSF4 mutations. Hum Genet 2005;**117**(5):452-9 doi: 10.1007/s00439-005-1309-9[published Online First: Epub Date]|.
19. Jamieson RV, Perveen R, Kerr B, et al. Domain disruption and mutation of the bZIP transcription factor, MAF, associated with cataract, ocular anterior segment dysgenesis and coloboma. Hum Mol Genet 2002;**11**(1):33-42
20. Shiels A, Bennett TM, Knopf HL, et al. CHMP4B, a novel gene for autosomal dominant cataracts linked to chromosome 20q. Am J Hum Genet 2007;**81**(3):596-606 doi: 10.1086/519980[published Online First: Epub Date]|.
21. Shiels A, Bennett TM, Knopf HL, et al. The EPHA2 gene is associated with cataracts linked to chromosome 1p. Mol Vis 2008;**14**:2042-55
22. Xia XY, Li N, Cao X, et al. A novel COL4A1 gene mutation results in autosomal dominant non-syndromic congenital cataract in a Chinese family. BMC Med Genet 2014;**15**:97 doi: 10.1186/s12881-014-0097-2[published Online First: Epub Date]|.
23. Balta B, Erdogan M, Kiraz A, Korkmaz S, Agadayi A. Frequent Mutation in the FTL Gene Causing Hyperferritinemia Cataract Syndrome in Turkish Population Is c.-160A>G. Turk J Haematol 2019;**36**(1):25-28 doi: 10.4274/tjh.galenos.2018.2018.0194[published Online First: Epub Date]|.
24. Churchill A, Graw J. Clinical and experimental advances in congenital and paediatric cataracts. Philos Trans R Soc Lond B Biol Sci 2011;**366**(1568):1234-49 doi: 10.1098/rstb.2010.0227[published Online First: Epub Date]|.
25. Li J, Leng Y, Han S, et al. Clinical and genetic characteristics of Chinese patients with familial or sporadic pediatric cataract. Orphanet J Rare Dis 2018;**13**(1):94 doi: 10.1186/s13023-018-0828-0[published Online First: Epub Date]|.
26. Taylor RL, Handley MT, Waller S, et al. Novel PEX11B Mutations Extend the Peroxisome Biogenesis Disorder 14B Phenotypic Spectrum and Underscore Congenital Cataract as an Early Feature. Invest Ophthalmol Vis Sci 2017;**58**(1):594-603 doi: 10.1167/iovs.16-21026[published Online First: Epub Date]|.
27. Tan YQ, Tu C, Meng L, et al. Loss-of-function mutations in TDRD7 lead to a rare novel syndrome combining congenital cataract and nonobstructive azoospermia in humans. Genet Med 2017

doi: 10.1038/gim.2017.130[published Online First: Epub Date]|.
28. Zhao L, Chen XJ, Zhu J, et al. Lanosterol reverses protein aggregation in cataracts. Nature 2015;**523**(7562):607-11 doi: 10.1038/nature14650[published Online First: Epub Date]|.
29. Khan K, Rudkin A, Parry DA, et al. Homozygous mutations in PXDN cause congenital cataract, corneal opacity, and developmental glaucoma. Am J Hum Genet 2011;**89**(3):464-73 doi: 10.1016/j.ajhg.2011.08.005[published Online First: Epub Date]|.
30. Guo H, Tong P, Peng Y, et al. Homozygous loss-of-function mutation of the LEPREL1 gene causes severe non-syndromic high myopia with early-onset cataract. Clin Genet 2014;**86**(6):575-9 doi: 10.1111/cge.12309[published Online First: Epub Date]|.
31. Bremond-Gignac D, Bitoun P, Reis LM, Copin H, Murray JC, Semina EV. Identification of dominant FOXE3 and PAX6 mutations in patients with congenital cataract and aniridia. Mol Vis 2010;**16**:1705-11
32. Economou M, Batzios SP, Pecci A, et al. MYH9-related disorders: report on a patient of Greek origin presenting with macroscopic hematuria and presenile cataract, caused by an R1165C mutation. J Pediatr Hematol Oncol 2012;**34**(6):412-5 doi: 10.1097/MPH.0b013e318257a64b[published Online First: Epub Date]|.
33. Shalaby AK, Emery-Billcliff P, Baralle D, et al. Identification and functional analysis of a novel oculocerebrorenal syndrome of Lowe (OCRL) gene variant in two pedigrees with varying phenotypes including isolated congenital cataract. Mol Vis 2018;**24**:847-52
34. Javadiyan S, Craig JE, Souzeau E, et al. High-Throughput Genetic Screening of 51 Pediatric Cataract Genes Identifies Causative Mutations in Inherited Pediatric Cataract in South Eastern Australia. G3 (Bethesda) 2017;**7**(10):3257-68 doi: 10.1534/g3.117.300109[published Online First: Epub Date]|.
35. Boone PM, Yuan B, Gu S, et al. Hutterite-type cataract maps to chromosome 6p21.32-p21.31, cosegregates with a homozygous mutation in LEMD2, and is associated with sudden cardiac death. Mol Genet Genomic Med 2016;**4**(1):77-94 doi: 10.1002/mgg3.181[published Online First: Epub Date]|.
36. Traverso M, Assereto S, Gazzerro E, et al. Novel FAM126A mutations in hypomyelination and congenital cataract disease. Biochem Biophys Res Commun 2013;**439**(3):369-72 doi: 10.1016/j.bbrc.2013.08.077[published Online First: Epub Date]|.
37. Aldahmesh MA, Khan AO, Mohamed JY, Alghamdi MH, Alkuraya FS. Identification of a truncation mutation of acylglycerol kinase (AGK) gene in a novel autosomal recessive cataract locus. Hum Mutat 2012;**33**(6):960-2 doi: 10.1002/humu.22071[published Online First: Epub Date]|.
38. Muller M, Bhattacharya SS, Moore T, et al. Dominant cataract formation in association with a vimentin assembly disrupting mutation. Hum Mol Genet 2009;**18**(6):1052-7 doi: 10.1093/hmg/ddn440[published Online First: Epub Date]|.
39. Yuan C, Han T, Su P, et al. A novel MIP mutation in a Chinese family with congenital cataract. Ophthalmic Genet 2018;**39**(4):473-76 doi: 10.1080/13816810.2018.1484930[published Online First: Epub Date]|.
40. Zhang XH, Da Wang J, Jia HY, et al. Mutation profiles of congenital cataract genes in 21 northern Chinese families. Mol Vis 2018;**24**:471-77
41. Ponnam SP, Ramesha K, Tejwani S, Matalia J, Kannabiran C. A missense mutation in LIM2 causes autosomal recessive congenital cataract. Mol Vis 2008;**14**:1204-8
